# Functional Characterization of a Novel Heterozygous Mutation in the Glucokinase Gene That Causes MODY2 in Chinese Pedigrees

**DOI:** 10.3389/fendo.2021.803992

**Published:** 2021-12-09

**Authors:** Feng Jiang, Jing Yan, Rong Zhang, Xiaojing Ma, Yuqian Bao, Yujuan Gu, Cheng Hu

**Affiliations:** ^1^ Department of Endocrinology, Shanghai Diabetes Institute, Shanghai Key Laboratory of Diabetes Mellitus, Shanghai Clinical Center for Diabetes, Shanghai Jiao Tong University Affiliated Sixth People’s Hospital, Shanghai, China; ^2^ Department of Endocrinology, Affiliated Hospital of Nantong University, Jiangsu, China; ^3^ Department of Endocrinology, Fengxian Central Hospital Affiliated to Southern Medical University, Shanghai, China

**Keywords:** glucokinase, MODY, mutation, A259T, mechanism

## Abstract

**Background:**

Glucokinase (GCK) plays a central role in glucose regulation. The heterozygous mutations of *GCK* can cause a monogenic form of diabetes, maturity-onset diabetes of the young (MODY) directly. In our study, we aimed to explore the mechanism of the novel mutation *GCK* p.Ala259Thr leading to glucokinase deficiency and hyperglycemia.

**Methods:**

Thirty early-onset diabetes pedigrees were referred to whole exome sequencing for novel mutations identification. Purified wild-type and mutant GCK proteins were obtained from *E.coli* systems and then subjected to the kinetic and thermal stability analysis to test the effects on GCK activity.

**Results:**

One novel missense mutation *GCK* p.Ala259Thr was identified and co-segregated with diabetes in a Chinese MODY2 pedigree. The kinetic analysis showed that this mutation result in a decreased affinity and catalytic capability for glucose. The thermal stability analysis also indicated that the mutant protein presented dramatically decreased activity at the same temperature.

**Conclusion:**

Our study firstly identified a novel MODY2 mutation p.Ala259Thr in Chinese diabetes pedigrees. The kinetic and thermal stability analysis confirmed that this mutation caused hyperglycemia through severely damaging the enzyme activities and protein stability.

## Introduction

Maturity Onset Diabetes of the Young (MODY) is a heritable and heterogeneous group of monogenic diabetes mellitus that are characterized by autosomal dominant inheritance, early onset and beta cell dysfunction. Mutations in at least 14 different genes (*HNF4A, GCK, HNF1A, PDX1, HNF1B, NEUROD1, KLF11, CEL, PAX4, INS, BLK, ABCC8*,* KCNJ11* and *APPL1*) have been shown to cause MODY subtypes 1–14 (1–3).

The *GCK* gene (7p15.3–p15.1) encodes the glucokinase (GCK) enzyme, which is a rate-limiting enzyme of glycolysis that is responsible for phosphorylating glucose to glucose-6-phosphate. GCK has unique kinetic characteristics, including a low affinity for glucose (S_0.5 =_ 5-8 mmol/L) (4), cooperativity with its glucose substrate (Hill coefficient, h=1.7), and a lack of inhibition by its product glucose-6-phosphate (G-6-P). In pancreatic β cells, GCK maintains glucose homeostasis through regulating glucose-stimulated insulin secretion in response to the intracellular glucose concentration (5–7). In the liver, GCK stimulates glucose disposal and glycogen storage (8). In addition, the crystal structures of human GCK present both active and inactive forms according to the glucose levels. Katama and colleagues revealed that GCK had a small and large domain that were separated by a deep cleft; these domains undergo a large conformational change through rotation of the small domain, which is induced by binding to glucose (9, 10).

Given its central role in glucose regulation, mutations in the gene encoding glucokinase can cause both hyper- and hypoglycaemia. Heterozygous activating GCK mutations can cause persistent hyperinsulinaemic hypoglycaemia of infancy (PHHI) (11). Furthermore, homozygous inactivating GCK mutations leading to complete GCK deficiency present as permanent neonatal diabetes mellitus (12), whereas heterozygous inactivating mutations are the underlying causes of MODY2 (13).

MODY is the most common type of monogenic diabetes, accounting for 2% to 5% of all diabetes cases in Europe (14). Previous studies indicate that *GCK*-MODY2, *HNF1A*-MODY3, *HNF4A*-MODY1 and *HNF1B*-MODY5 account for more than 95% cases of MODY in Caucasians, but only account for just 10–20% of MODY cases in Asia (including China, Japan and Korea) (15). An epidemiological investigation in Chinese hyperglycemia pedigrees that fulfilling the clinical diagnostic criteria for MODY show that the MODY subtype detection rate was 18.42% for GCK (16). Heterozygous mutations in *GCK* lead to decreased glucokinase activity and thus deficient sensitivity to glucose in β cells and impaired glycogen synthesis in the liver (17). GCK/MODY2 occurs with a mild non-progressive hyperglycaemia, which generally is asymptomatic and develops without an increased risk of late complications, such as diabetic retinopathy or nephropathy (18, 19). Due to the unapparent symptoms, MODY2 is often misdiagnosed and treated inappropriately. However, a molecular genetic diagnosis can change the management, since patients with *GCK* mutations rarely require pharmacological treatment. Thus, a correct genetic diagnosis is important for guidance of the prediction of asymptomatic relatives and personalized treatment for those with diabetes. To date, although more than 600 different GCK/MODY2 mutations have been reported, including nonsense, missense, and frameshift mutations, less than 20% of these mutations have been functionally characterized (20–22). Pathophysiological studies on naturally occurring GCK missense mutations will provide further clues to help elucidate the mechanisms of glycaemic disorders and investigate the biological characteristics of this enzyme.

In this study, we report the novel *GCK* missense mutation Ala259Thr, which co-segregates with diabetes in Chinese MODY families for the first time. This mutation, which alters alanine to threonine at the 259^th^ amino acid, is located proximal to the glucose-binding site but has not been investigated biochemically. Herein, we discovered that the Ala259Thr mutation exerted effects on the catalytic activity and protein thermostability of glucokinase.

## Materials and Methods

### Subjects

The retrospective study included 30 early-onset diabetes pedigrees referred for genetic testing. All pedigrees were clinically diagnosed with MODY according to the following classic criteria (23, 24): a family history diabetes for at least two consecutive generations, early-onset of diabetes before the age of 25 years, no need for insulin therapy, and negative for type 1 diabetes antibodies. The diagnosis was made based on an oral glucose tolerance test (OGTT). The fasting blood glucose (FPG), 2h blood glucose (2h PG), fasting insulin (FINS), 2h insulin (2h-INS) and glycated hemoglobin (HbA1c) levels were measured in all family members available for testing.

The study was performed according to the Declaration of Helsinki and was approved by our institutional review boards. Informed consent was obtained from all family members.

### Identification of Glucokinase Gene Mutations by Whole Exome Sequencing

Genomic DNA was extracted from peripheral lymphocytes using a Qiagen DNA extraction kit (Qiagen, Frankfurt, Germany). Whole exome sequencing was performed to explore novel mutation and direct sequencing was used to validate the positive mutation. The coding regions of exons 1a-10 and the intron-exon boundaries of the GCK gene were amplified by PCR using self-designed primers (**Table 1**). PCR products were purified using QIAquick PCR purification columns (Qiagen, Frankfurt, Germany), and both strands were sequenced using the BigDye Terminator Cycle Sequencing Kit (Applied Biosystems, CA, UK) according to the manufacturer’s recommendations.

**Table 1:** Amplification and sequencing primers for the *GCK* exons.

Exon	Forward primers (3’-5’)	Reverse primers (3’-5’)	Product length (bp)
Exon-1a	TCCACTTCAGAAGCCTACTG	GTTTGAGCCTCAGAATCTGA	195
Exon-1b	AGCAGGCAGGAGCATCTCTG	CTTTGCACTGGGAGAGCAGC	149
Exon-1c	GAACTCGGGCCTCACATG	GGATTGTTAGGACAGCCTG	252
Exon 2	TGTGCAGATGCCTGGTGA	CACTCCCAGACTCACAGCC	343
Exon 3	TAATATCCGGGCTCAGTCACCT	CAAGGCCATGCAGGATCTCAG	298
Exon 4	TAGCTTGGCTTGAGGCCGTG	CCAGAGGAACTCTGCCTTCA	272
Exon 5	GCAGCCACGAGGCCTATCTC	CAGCACTGCCTGCCTTTCTC	195
Exon 6	CCAGCACTGCAGCTTCTGTG	CTTCCAGACTGCTGAGGCTC	176
Exon 7	AGCCGCCTTTCCATTGTT	AAAAGCAAACTGACAATCCGTT	451
Exon 8	CCTCCCTCGTGCCTGCTGAT	ACTTGGTCTCAGGGCGACG	279
Exon 9	ACTGTCGGAGCGACACTCAG	TGCGGTTCCCAAGCTCCAAG	367
Exon 10	CGCCCGGTAATGAATGTGG	CCACAGCACCCAGGCTCCAT	269

### Production and Purification of Recombinant Wild-Type and Mutant Glucokinase

Recombinant human wild-type liver GCK was constructed with a His tag at the NH2 terminal and ligated into the pET-28a(+) vector. The Ala259Thr mutation was generated based on the His-GCK construct by PCR using a kit (QuikChange II Site-Directed Mutagenesis Kit, Stratagene, CA, USA). The following oligonucleotide was used to generate the Ala259Thr mutation: forward primer (5’ CGAGTGGGGCACCTTCGGGGACTCCGGCGAGCTGGACGAGTT 3’) and reverse primer (TCCCCGAAGGTGCCCCACTCGGTATTGACGCACATGCGGCCCT). The wild-type and mutant GCK sequences were verified using the ABI 3500xl DNA sequencer (Applied Biosystems, USA). The wild-type and mutant GCKs with His tags were transformed into *E. coli* (BL21-CodonPlus (DE3)-RIPL chemically competent cells) and then purified from 30-g cell pellet. Two-step affinity chromatography was used with Ni-NTA beads to bind the fusion protein, which was eluted with Ni-NTA and loaded onto the Superdex™ 200 16/60 column. Both the wild-type and mutant His-GCK purified proteins showed a single band on SDS-PAGE gels. The purified proteins were quantified using the Bradford method (Bradford Protein Assays, Thermo Fisher Scientific, USA) using standard methods and stored at -80°C in 30% glycerol, 5 mmol/L glutathione, 5 mmol/L dithiothreitol (DTT), 200 mmol/L KCl, and 50 mmol/L Tris buffer (pH 7.4).

### Kinetic Analysis

GCK activity was measured spectrophotometrically based on the ADP-Glo™ Kinase Assay (Promega, USA). The luminescent signal generated is proportional to the ADP concentration produced and is correlated with the kinase activity. Kinetic parameters were also determined according to the assay as follows. First, standard ATP/ADP mixtures representing different conversion percentages were prepared to generate the standard curve for conversion of ATP to ADP. Second, ten serial two-fold dilutions of glucose in the assay buffer (final concentration starting from 200 mM) in the presence of 1 mM ATP were generated to determine the optimal glucose concentration when the half maximal velocity (V_max_) of the reaction was reached. The assay buffer contained 50 mM Tris, 100 mM KCl and 10 mM MgCl_2_ (pH 7.5). GraphPad Prism 7.0 (GraphPad Software, La Jolla, CA, USA) was used to calculate the glucose-K_m_ (S_0.5_), glucose-K_cat_, ATP-K_m_, ATP-K_cat_, Hill coefficient (h) and inflection point of glucose. The relative activity index and the glucose concentration at the inflection point were also calculated.

### Thermal Stability Analysis

The thermal stability of the mutant and wild-type His-GCK enzymes was assessed using the ADP-Glo™ Kinase Assay with 3 mM (for the wild-type)/11 mM (for the mutant) glucokinase and 1 mM ATP. The enzymes were incubated in a water bath at 25, 30, 35, 40, 45, 50, 55, and 60°C for 30 min or 50°C for 0, 5, 10, 15, 20, 25, and 30 min. Luminescence was measured to represent the glucokinase activity as described above.

### Statistical Analysis

All results are presented as the mean ± SD. Student’s two-tailed unpaired t test was used to assess differences between groups. The Mann-Whitney test was used to evaluate differences in clinical parameters between the mutants and non-mutants. The statistical analyses were performed using SAS 8.0 (SAS institute, Cary, NC, USA). A two-tailed p value less than 0.05 was considered significant.

## Results

### Identification of a Novel Missense Mutation in the *GCK* Gene

**Figure 1:**
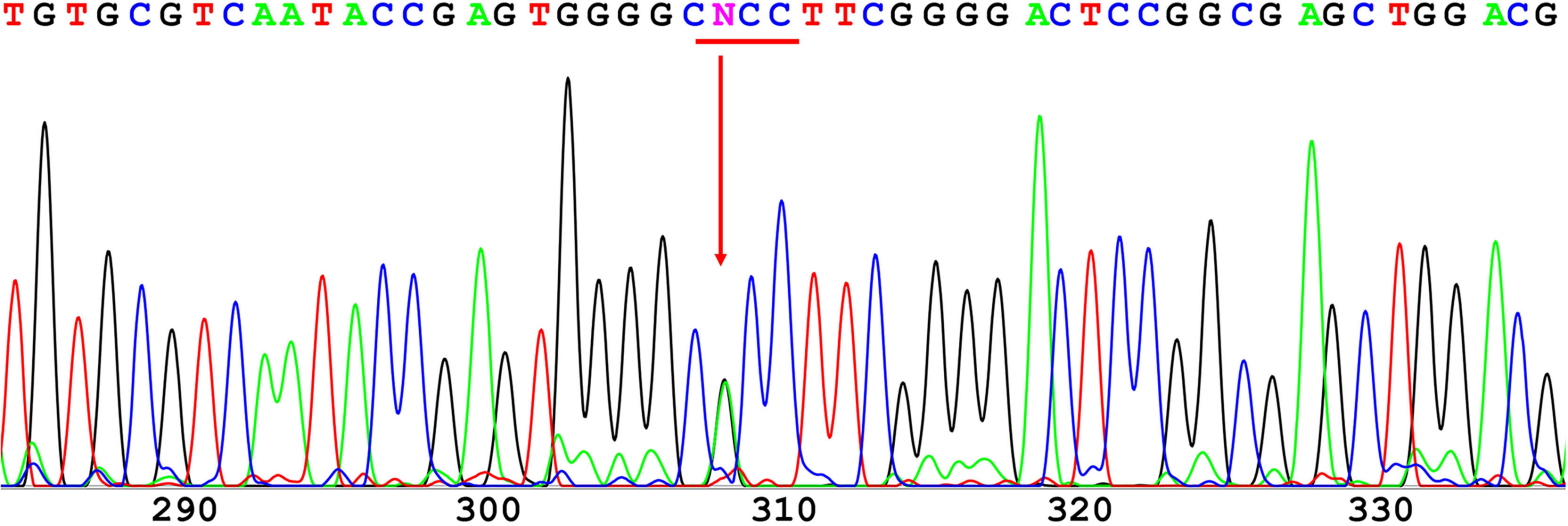
Direct sequencing of GCK exon 7. A novel heterozygous missense mutation was found in codon 259 and resulted in a substitution of alanine (GCC) to threonine (ACC).

The 12 exons of the *GCK* gene were scanned for the validation of mutations using direct sequencing for each of the affected families. A novel heterozygous missense mutation in GCK gene exon 7 (codon 259 GCC➔ACC; **Figure 1**) resulting in an amino acid substitution (Ala^259^➔Thr) was identified in the proband. The same mutation was also identified in the proband’s father and grandfather. Conversely, this mutation was not found in the four unrelated healthy individuals used as controls.

### Clinical Profiles of the Patients

**Figure 2:**
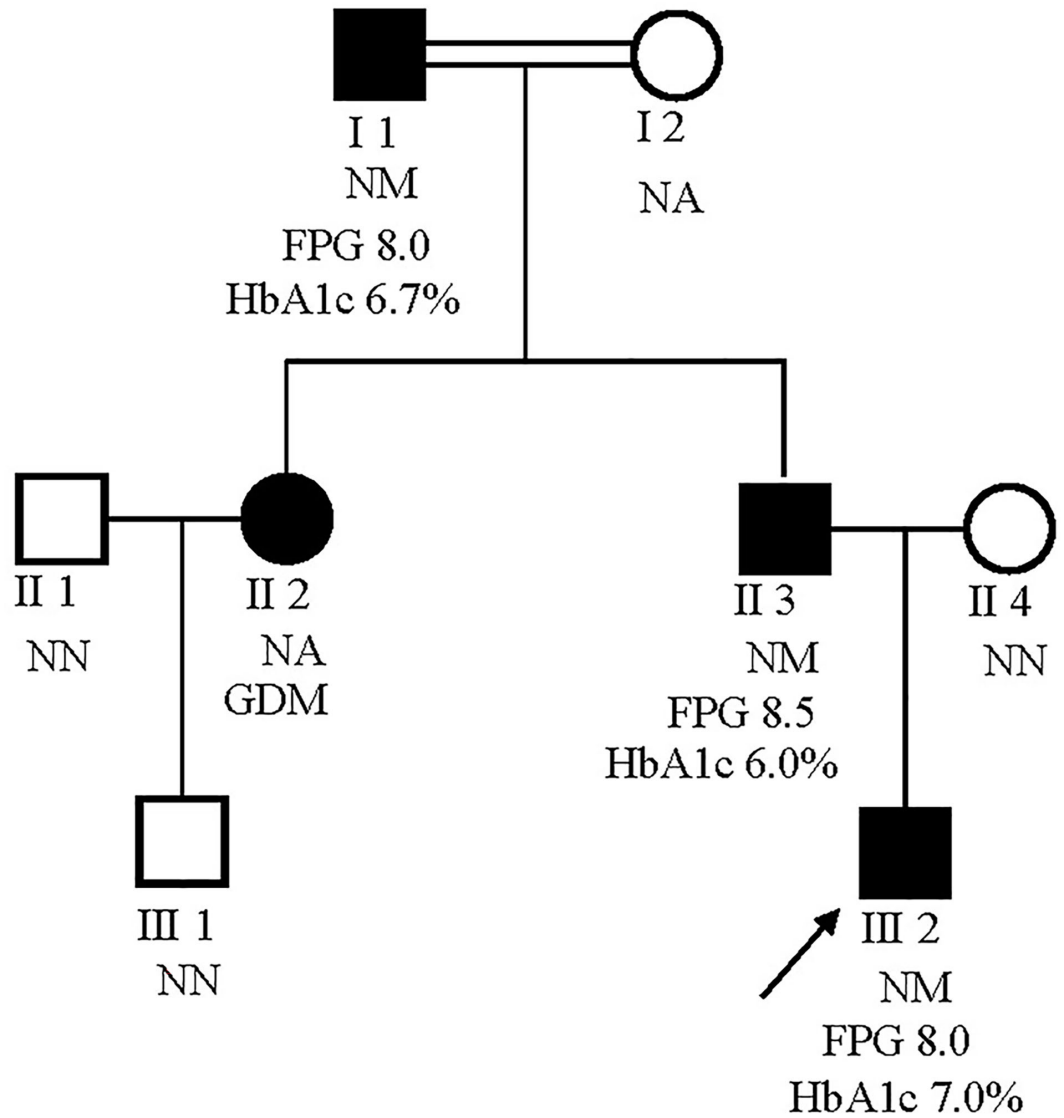
Pedigrees for a MODY2 family showing co-segregation of the Ala259Thr GCK mutation with diagnosed diabetes. Circles represent females, and squares represent males. Black shaded shapes represent diabetic individuals. The number under each member represents the sample identifier. NN, no mutation; NM, heterozygous for Ala259Thr mutation; NA, not available for testing. The FPG and HbA1c values are shown where available.

The male proband (III:2) was diagnosed with diabetes at 5 years of the age and presented with fasting hyperglycemia. Biochemical studies showed elevated fasting plasma glucose (FPG) (8.0 mmol/L), 2h plasma glucose (2h PG)(12.8 mmol/L) after oral glucose tolerance test (OGTT), and glycatedhemoglobin A1c (HbA1c) (7%). In contrast, fasting insulin (FINS) and 2h insulin (2h-INS) decreased (FINS: <0.2 uU/ml, 2h-INS: 2.23 uU/ml). The proband’s father (II:3) and grandfather (I:2) were diagnosed with fasting hyperglycaemia at the ages of 33 and 47 years, respectively, during routine screening (FGP=7.8 and 9 mmol/L, respectively). The HbA1c level was elevated in proband’s grandfather (7.5%), but was normal in proband’s father (6%). Both FINS and 2h-INS were normal in proband’s father and grandfather. The proband’s paternal aunt (II:2) presented with gestational diabetes mellitus (GDM) during her first pregnancy according to the previous medical history. But it is unavailable for us to get her biochemical results. None of the diabetic patients received hypoglycemic drugs. The rest of the family were normal glucose regulation (NGT) individuals with normal blood glucose and insulin levels (**Figure 2**).

### Production of Recombinant Wild-Type and Mutant Glucokinase

The recombinant wild-type and mutant enzymes were expressed in an *E. coli* system (BL21(DE3)-Gold). Nine preparations of the His-fusion protein were purified with a Ni-NTA column, thrombin digestion and Superdex™ 200 column, with yields of 11.5 mg/L and 10 mg/L for the wild-type and mutant proteins, respectively. All His-GCK proteins were proven to be essentially pure based on the presence of a single band at 75 kDa on a SDS-PAGE gel.

### Kinetic Analysis

**Figure 3:**
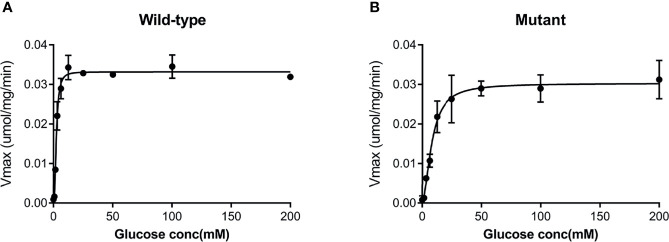
Response curve of GCK for a series of glucose concentrations. **(A)** Wild-type GCK, **(B)** mutant GCK. The Ala259Thr mutation showed a lower maximum reaction velocity than the wild-type GCK, indicating a lower affinity for glucose.

**Figure 4:**
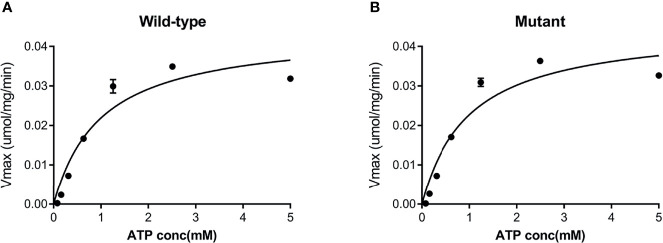
Response curve of GCK for a series of ATP concentrations. **(A)** Wild-type GCK, **(B)** mutant GCK. The wild-type GCK and Ala259Thr mutation showed a similar maximum reaction velocity, indicating a similar affinity for the second substrate (ATP).

Both the purified wild-type and mutant GCKs were subjected to kinetic analysis using glucose and ATP as substrates. The response curves of GCK for a series of glucose or ATP concentrations, which indicate the affinity of GCK for the substrates, are shown in **Figure 3** (glucose as substrate) and **Figure 4** (ATP as substrate). The kinetic parameters, including the substrate affinities (S_0.5_ for glucose and ATP-K_m_ for ATP), catalytic constants (glucose-K_cat_ and ATP-K_cat_, respectively), Hill coefficients and inflection points of glucose, are shown in **Table 1**. The Ala259Thr mutant showed a 2.3-fold lower affinity for glucose (S_0.5_ for mutant and wild-type, 2.42 ± 0.14 vs. 7.95 ± 0.92, p=0.0005), but a similar affinity to the second substrate ATP (ATP-Km for mutant and wild-type, 1.00 ± 0.25 vs. 1.00 ± 0.24, p>0.05). Moreover, the glucose-K_cat_ was significantly higher for the wild-type than for the mutant (33.2 ± 0.6 vs. 30.3 ± 1.2, p=0.0201), whereas the ATP-K_cat_ was similar between the two groups (43.8 ± 3.9 vs. 45.2 ± 4.1, p=0.6903), indicating a decreased catalytic capability of the mutant enzyme for glucose but not for ATP. In addition, the Ala259Thr mutant presented a significantly higher inflection point than the wild-type enzyme (3.78 ± 0.44 vs. 1.69 ± 0.10, p=0.0013), indicating a greater demand for glucose when the maximal velocity was reached in the reaction system. Finally, the mutant showed a lower Hill coefficient than the wild-type enzyme (2.44 ± 0.29 vs. 1.75 ± 0.30, p=0.0457), implying decreased cooperativity for the glucose substrate (**Table 2**).

**Table 2:** Kinetic parameters of the wild-type and mutant forms of His-GCK.

	S_0.5_ (mM)*	ATP-Km (mM)	Glucose-K_cat_*	ATP-K_cat_	Hill coefficient (h) *	Inflection point (mM)*
Wild-type	2.42 ± 0.14	1.00 ± 0.24	33.2 ± 0.6	43.8 ± 3.9	2.44 ± 0.29	1.69 ± 0.10
Mutant	7.95 ± 0.92	1.00 ± 0.25	30.3 ± 1.2	45.2 ± 4.1	1.75 ± 0.30	3.78 ± 0.44

Means ± SD are given. The S0.5 value, ATP-Km, Kcat value for glucose (Glucose-Kcat), ATP (ATP-Kcat), Hill coefficient and inflection point were obtained from the allosteric sigmoidal equation. Note that the Hill coefficient (h) is unitless.

*p < 0.05.

### Thermal Stability Analysis

**Figure 5:**
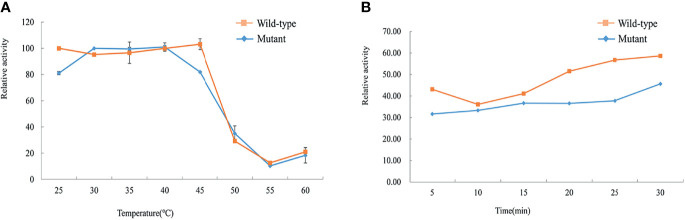
Assessment of thermal instability of the wild-type and mutant GCK proteins. Squares represent wild-type GCK, and circles represent mutant GCK. **(A)** Relative activity of the wild-type and mutant GCKs at different temperatures in the kinase reaction. **(B)** Stability of the wild-type and mutant GCKs at 50°C in the kinase reaction.

The thermostability tests of the wild-type and mutant His-GCK enzymes were performed at different temperatures to investigate protein stability, which was also a key determinant of enzyme function. The enzyme activity of wild-type GCK was stable under a temperature of 45°C with a sharp decline at 50°C (**Figure 5A**). In contrast, the Ala259Thr mutant maintained activity under a temperature of 40°C but decreased dramatically at 45°C (**Figure 5A**). However, the mutant maintained decreased enzyme activity similar to the wild-type enzyme at 50°C that was maintained for 30 min (**Figure 5B**). The statistical analyses were performed with SAS 8.0 (SAS Institute, Cary, NC, USA). A two-tailed p value <0.05 was considered significant.

## Discussion

MODY is an autosomal dominant form of diabetes that is characterized by early onset, pancreatic dysfunction and a non-insulin-dependent diabetes status. Heterozygous mutations in *GCK* were first recognized as the intrinsic cause of MODY2 in 1992 (13, 25). *HNF1A*, *GCK*, *HNF4A*, and *HNF1B are the most common types* in Europeans. MODY2 accounts for approximately 80% of MODY patients in Span (22), 38-86% in Italy (26–28), 56% in France (29) and 32% in United Kingdom (30). However, MODY2 is rarely reported in Asian patients, with a prevalence of 1% in Japanese (31), 2% in Korean (32) and 1-4% in Hong Kong Chinese patients (33, 34).

In our study, we recruited 30 early-onset diabetes pedigrees for genetic testing and discovered the novel mutation Ala259Thr in *GCK*, which was accompanied by hyperglycaemia and was in accordance with autosomal dominant inheritance. Three of the four diabetic patients in this pedigree were characterized by mild hyperglycaemia. The patients with Ala259Thr mutations did not require treatment, but could be managed with diet or exercise. Although we could not confirm whether the remaining female patient was a mutation carrier, since her DNA sample was not available, this patient was definitely diagnosed with GDM during her first pregnancy and returned to a normal glucose level after delivery. Therefore, this family was confirmed to be a MODY2 pedigree linked to a novel *GCK* p.Ala259Thr mutation.

Different MODY2 mutations have been reported to impair GCK function through different mechanisms, including kinetics, enzymatic activity or protein thermostability (18, 22, 35–38). Therefore, we investigated the functional characteristics of the Ala259Thr recombinant protein to elucidate the potential mechanism resulting in hyperglycaemia. In the kinetic analysis, Ala259Thr presented a higher S_0.5_ value and inflection point, which indicated that a high glucose concentration was required to achieve the V_max_. Moreover, the lower Hill coefficient and K_cat_ of the Ala259Thr mutation revealed a decreased affinity and catalytic activity when glucose was used as the substrate. However, when ATP was used as the substrate, the K_m_-ATP and K_cat_ showed no significant differences between the mutant and wild-type recombinant proteins, which suggested that the Ala259Thr mutation affected the binding or catalytic capacity for glucose but not ATP. No mutation was reported in the same position previously, but the nearby mutations p.Trp257Arg and p.Gly261Arg presented decreased K_cat_ values (39, 40).

Most of the GCK mutations reported were found to be kinetically inactive, with alterations of one or more kinetic parameters (22, 35, 40). However, kinetic inactivation may not be the only factor that causes hyperglycaemia. Different MODY2 mutations have been reported to impair GCK function through different mechanisms, including kinetics, enzymatic activity or protein thermostability (36, 37). In our study, we also performed a thermal analysis. The wild-type GCK recombinant protein was stable under a temperature of 45°C, whereas the mutant protein presented dramatically decreased activity at the same temperature. Since the temperature for protein thermostability exceeds the normal temperature of the human body, the effect of thermal stability on hyperglycemia remained to be confirmed. As for the investigation of enzyme function, biochemical experiments are still the first choice in most literature reports since researchers can directly obtain recombinant protein and perform kinetic and thermal stability analysis to evaluate the activity and stability of the enzyme. However, it might be better to perform functional experiments *in vivo* to demonstrate the mechanism that the mutation contributing to hyperglycemia.

In addition, the glucokinase regulatory protein (GKRP) could act as a competitive inhibitor of glucose and regulate GCK activity through protein-protein interactions (41). Posttranslational regulation of GCK could also influence GCK activation (14). For example, cytoplasmic Ca(2+) levels may regulate GCK activation and therefore glucose metabolism and insulin secretion (42). The GCK-R369P and GCK-V367M mutations could impair glucose-stimulated insulin secretion through posttranslational regulation of GCK S-nitrosylation (14). However, whether these mechanisms participate in *GCK* p.Ala259Thr activity needs to be elucidated.

In the present study, we identified the novel mutation GCK p.Ala259Thr that co-segregated with diabetes in a Chinese MODY2 pedigree. Our study illustrated that the GCK p.Ala259Thr mutation had an immediate impact on the kinetic inactivity and thermal instability of the GCK enzyme, which led to hyperglycaemia in the mutation carriers of this pedigree. Other potential mechanisms, such as posttranslational regulation or crystal structure crystallographic analysis, need to be assessed in future studies.

## Data Availability Statement

The data analyzed in this study is subject to the following licenses/restrictions: Datasets consist of data routinely recorded in clinical practice. Requests to access these datasets should be directed to Cheng Hu, alfredhc@sjtu.edu.cn.

## Ethics Statement

Ethical approval was granted by the Institutional Review Board of Shanghai Jiao Tong University Affiliated Sixth People’s Hospital. Written informed consent to participate in this study was provided by the participants’ legal guardian/next of kin.

## Author Contributions

CH and YG contributed to the study design, acquisition and interpretation of data, reviewed and edited the manuscript. FJ focused on the biological experiments, analysis and interpretation of data, drafted and edited the manuscript. JY contributed to the biological experiments. RZ, XM, and YB contributed to the pedigree collection, genetic testing and clinical diagnosis. All authors contributed to the article and approved the submitted version.

## Funding

This work was supported by the National Science Foundation of China (NSFC) (31500955, 81800702), the Shanghai Outstanding Academic Leaders (20XD1433300) and the Interdisciplinary Program of Shanghai Jiao Tong University (YG2021ZD20) and Nantong Municipal Science and Technology Project (MS22019005).

## Conflict of Interest

The authors declare that the research was conducted in the absence of any commercial or financial relationships that could be construed as a potential conflict of interest.

## Publisher’s Note

All claims expressed in this article are solely those of the authors and do not necessarily represent those of their affiliated organizations, or those of the publisher, the editors and the reviewers. Any product that may be evaluated in this article, or claim that may be made by its manufacturer, is not guaranteed or endorsed by the publisher.
